# Autonomous dental implant robotics for transcrestal maxillary sinus floor elevation: workflow development and clinical trueness evaluation

**DOI:** 10.1186/s12903-026-08381-9

**Published:** 2026-05-22

**Authors:** Jiakang Qian, Wang Zi-Zheng, Xinya Teng, Xiaohui Qu, Ying Qu, Derong Zou

**Affiliations:** 1 Department of Stomatology, Shanghai Sixth People’s Hospital Affiliated to Shanghai Jiao Tong University School of Medicine, No. 600, Yishan Road, Xuhui District, Shanghai, 200233 PR China; 2 Department of Stomatology, Changsha Medical University, Changsha, 410219 Hunan P. R. China

**Keywords:** Dental implantation, Endosseous, Surgery, Computer-assisted, Robotic surgical procedures, Sinus floor augmentation

## Abstract

**Purpose:**

To evaluate the trueness and safety of an autonomous dental implant robotic system (ADIRS) for transcrestal maxillary sinus floor elevation (TSFE), to compare outcomes between flat and sloped sinus floor morphologies, and to summarize a morphology-specific operative workflow.

**Methods:**

This single-center retrospective cohort included 11 patients (12 implants) who underwent ADIRS-assisted TSFE between June 2024 and June 2025. Planned and postoperative cone-beam computed tomography datasets were superimposed to quantify global deviations at the coronal (platform) and apical levels and angular deviation. Intraoperative endoscopic inspection and the Valsalva maneuver were used to detect Schneiderian membrane perforations.

**Results:**

Mean global deviations at the coronal and apical levels were 0.58 ± 0.38 mm (range 0.17–1.63) and 0.64 ± 0.35 mm (range 0.37–1.62), respectively, and the mean angular deviation was 1.19 ± 0.50° (range 0.56–2.43). No Schneiderian membrane perforations or other intraoperative or postoperative complications occurred. Trueness metrics did not differ significantly between the flat (*n* = 7) and sloped (*n* = 5) groups.

**Conclusion:**

ADIRS-assisted TSFE achieved submillimeter positional trueness with reliable protection of the sinus membrane in posterior maxillary implant placement, supporting its use as a standardized, minimally invasive sinus augmentation protocol.

**Supplementary Information:**

The online version contains supplementary material available at 10.1186/s12903-026-08381-9.

## Background

Maxillary sinus floor elevation is a key procedure for overcoming insufficient bone height in the posterior maxilla. Among the available approaches, transcrestal maxillary sinus floor elevation (TSFE) is widely adopted in cases with mild to moderate ridge resorption [1–3]. However, conventional TSFE remains highly operator dependent, which leads to variable surgical accuracy and a non-negligible risk of complications such as sinus membrane perforation, postoperative headache, and swelling [1, 2, 4, 5]. Because of the anatomical characteristics of the Schneiderian membrane and the limited intraoperative visibility, TSFE is essentially a semi-blind procedure, thereby increasing the technical difficulty for clinicians [1, 6, 7].

In recent years, robot-assisted surgery has progressed rapidly in implant dentistry, demonstrating advantages in single-tooth, full-arch, and complex rehabilitations [8–11]. Several dental implant robotic systems, including Yomi, DentRobot, and Remebot, have been introduced into clinical practice [8, 10, 12]. Among these systems, semi-active robotic platforms integrate robotic positioning, haptic feedback, and vision guidance to execute the preplanned trajectory and to constrain drilling depth and angulation. Near-real-time feedback on drilling resistance at the sinus floor assists the surgeon in estimating membrane proximity and may enhance intraoperative safety [9, 12, 13]. Early clinical studies of semi-active, robot-assisted TSFE have reported submillimetric global coronal and apical deviations of approximately 0.5–0.8 mm and angular deviations of approximately 1.2–2.2°, together with low rates of intraoperative complications [8, 10, 12–14].

Despite the growing clinical use of semi-active dental robotic systems, the operative workflow of each platform remains insufficiently standardized [10, 11]. Among these emerging platforms, the autonomous dental implant robotic system (ADIRS), built on the YakeBot architecture, enables fully digital, end-to-end surgical execution and reduces the need for intraoperative manual intervention [15, 16]. However, the current clinical evidence is predominantly based on Remebot-assisted procedures, whereas data on ADIRS for TSFE are limited and mainly exploratory. The clinical feasibility, achievable trueness, and safety profile of ADIRS-assisted TSFE therefore require further evaluation in real-world patients [11].

Sinus floor morphology is another key determinant of TSFE difficulty and the risk of Schneiderian membrane perforation. Flat sinus floors are generally more controllable and are associated with lower perforation and complication rates than sloped sinus floors [3, 5, 6, 12]. Nevertheless, no study has systematically described morphology-specific TSFE workflows based on ADIRS or investigated whether flat and sloped sinus floors differentially affect the trueness of robot-guided implant placement in TSFE.

Accordingly, in this study we integrated ADIRS with self-designed sinus elevation instruments to evaluate the trueness and complication profile of robot-guided TSFE. We further summarized morphology-specific operative strategies and compared outcomes between flat and sloped sinus floor groups, with the aim of establishing a standardized and generalizable workflow for ADIRS-assisted TSFE.

## Materials and methods

### Study design and participants

This single-center retrospective cohort included consecutive patients who underwent ADIRS-assisted TSFE at the Department of Stomatology, Shanghai Sixth People’s Hospital, between June 2024 and June 2025. The study complied with the Declaration of Helsinki, adhered to the STROBE guidelines, and was approved by the institutional ethics committee (approval No. 2024–080). All participants provided written informed consent.

#### Inclusion criteria were as follows

(1) age ≥ 18 years; (2) good oral hygiene or hygiene improved to an acceptable level after basic periodontal therapy; (3) single or short-span edentulism in the posterior maxilla with preoperative CBCT showing a residual bone height (RBH) of 4–8 mm at the planned implant site, measured on coronal or sagittal slices along the implant centerline, with indication for TSFE; (4) maxillary sinus without clinically relevant inflammatory mucosal thickening, cystic lesion, prominent vascular variation, or bony obstacle that would interfere with instrument access during TSFE (e.g., Underwood’s septa); and (5) ability to undergo digital impression, ADIRS-guided surgery, and scheduled postoperative follow-up.

#### Exclusion criteria were as follows

(1) systemic diseases contraindicating routine oral implant surgery; (2) heavy smoking (> 10 cigarettes/day) or alcohol abuse; (3) poor oral hygiene or untreated active periodontitis; (4) RBH < 4 mm or > 8 mm at the planned implant site; and (5) pregnancy or lactation.

Because this retrospective cohort included all eligible patients within the study period, no a priori sample size calculation was performed. Details of the post hoc sensitivity analysis are provided in Sect. "Statistical analysis".

### Preoperative preparation

**Fig. 1:**
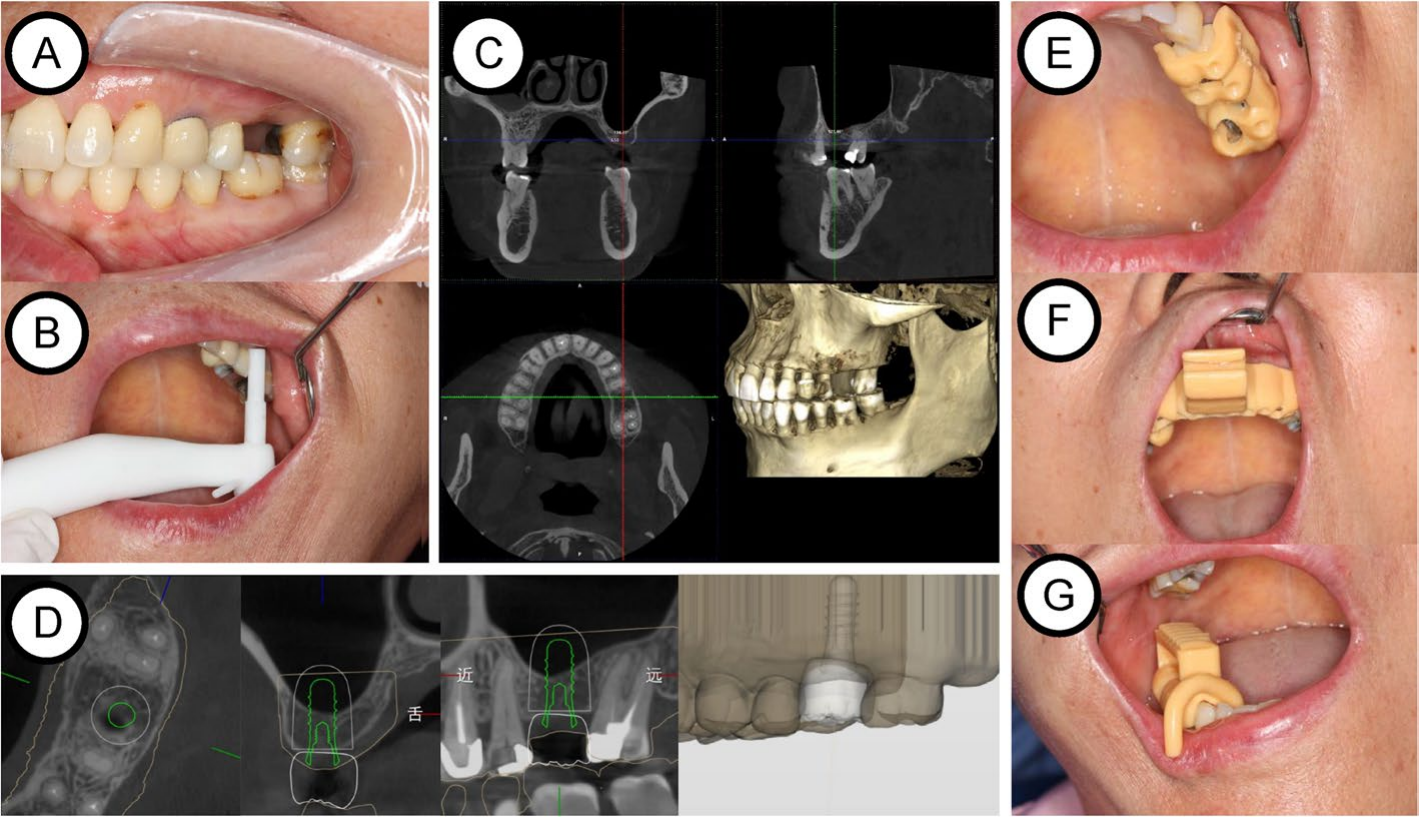
Preoperative preparation and data acquisition. **A** Intraoral view of the edentulous site. **B** Assessment of mouth opening. **C** Preoperative CBCT with multiplanar and 3D reconstruction. **D** Prosthetically driven implant planning in DentalNavi 3.0. **E** Intraoral try-in of the attachment for the posterior edentulous region. **F** Intraoral try-in of the attachment for the anterior region. **G** Mouth opener and suction tubing

All patients underwent routine oral examination (edentulous site, soft- and hard-tissue condition, occlusal relationship, interarch space) and basic laboratory tests (complete blood count, coagulation profile, liver and renal function, infectious-disease screening) to exclude surgical contraindications (Fig. 1A, B). Periodontal treatment was provided when necessary to ensure an acceptable level of oral hygiene.

The ADIRS (DRS-FT250, YakeRobot, Beijing, China) was checked preoperatively to ensure normal hardware and software performance. Patients underwent CBCT (Planmeca ProMax 3D Mid, Finland) in a standard position with the following parameters: voxel size 0.15 mm, 12.5 mA, 100 kV, and 8.0 s scanning time (Fig. 1C). An intraoral scanner (TRIOS, 3Shape, Denmark) was used to capture the maxillary and mandibular arches. CBCT and intraoral scan data were imported into the planning software (DentalNavi 3.0, YakeRobot) for data registration and prosthetically driven surgical design. A sinus floor with a nearly straight or gently curved contour and a height difference < 2.0 mm between the buccal and palatal ends (or the anterior and posterior ends in the sagittal plane) within the same slice was classified as flat. Sinus floors exhibiting a noticeable inclination toward the buccal or palatal side, or a height difference ≥ 2.0 mm, were classified as sloped. Implant dimensions were selected to meet prosthetic load requirements while prioritizing surgical safety by limiting the planned Schneiderian membrane elevation. When ridge width permitted, a 4.8 mm diameter implant with an 8 mm length was typically used, and a 10 mm tapered implant BLT RC 4.8*10 mm was considered in selected molar sites with relatively favorable residual bone height to enhance primary stability. When ridge width was limited, a 4.1 mm diameter implant was used and length was usually 10 mm, while a 4.1*8 mm implant was selected when minimizing elevation was critical to reduce perforation risk and when bone conditions and primary stability were considered sufficient to support functional loading. In the virtual environment, TSFE was simulated, the stepwise osteotomy path and the implant position and depth were planned, and a complete surgical plan was generated (Fig. 1D). A positioning guide with registration points was then designed and 3D-printed according to this plan. Depending on the identified sinus-floor morphology, the preplanned TSFE execution strategy was tailored as follows:

#### Flat sinus floors

Conventional drilling was planned to prepare the osteotomy, with the endpoint set 1 mm short of the sinus-floor cortical plate. The sinus floor milling instrument would then be applied, and the robotic system programmed to switch to automatic traction (auto-lift) mode with a force threshold of 20 N and a rotational speed of 150 rpm (to ensure a safety margin for the Schneiderian membrane). The instrument endpoint was set to be positioned at the superior border of the sinus-floor cortical plate, so that the cortical bone could be gradually milled. The protocol dictated that if the terminal force sensor detected a sudden pressure drop, the foot pedal must be released immediately. After confirmation of sinus membrane integrity, the sinus membrane elevator would be operated at 50 rpm to gently elevate the membrane, followed by the planned placement of CGF membranes and bone–collagen material. The implant would then be inserted into the planned position, with continuous recording and monitoring of insertion resistance.

#### Sloped sinus floors

**Fig. 2:**
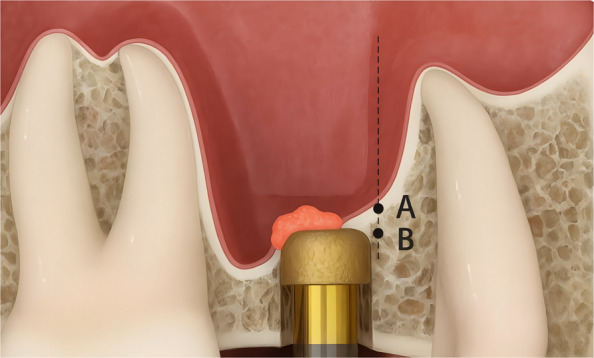
Drill insertion in a sloped sinus floor. Schematic illustrating the drill advancing to point B, with point A representing the final sinus floor elevation level and point B located 0.5 mm coronal to point A

Conventional drilling was planned to prepare the osteotomy until the cutting edge of the twist drill contacted the sinus-floor cortical bone. The Dentium Advanced Sinus Kit (DASK; Dentium, Korea) would then be used, and auto-lift mode programmed to activate with a force threshold of 30 N and a rotational speed of 150 rpm (maintaining a conservative safety margin for the Schneiderian membrane). The sinus-floor bone at one corner of the osteotomy was planned to be milled first to expose a small area of the Schneiderian membrane. A small Bio-Gide patch, covering approximately half of the osteotomy area, would be placed to protect the exposed membrane. The drill trajectory was planned to advance to point B, set 0.5 mm short of point A, to compensate for potential imaging error that might otherwise lead to premature cortical penetration, prior to probing the sinus-floor bone (Fig. 2). The drill would then be advanced to point A. The protocol specified that if cortical resistance persisted, the endpoint would be advanced 0.5 mm apically to facilitate controlled milling of the remaining sinus-floor cortical bone and reduce resistance. After reconfirming membrane integrity, the sinus membrane elevator would be used at 50 rpm to elevate the membrane. CGF membranes and bone–collagen material would then be planned for placement, and the implant inserted at the planned position with continuous monitoring of insertion resistance.

We performed a preoperative try-in of all surgical attachments. Through the observation window, we confirmed that the attachments adapted closely to the tooth surface without rocking. We also confirmed that mouth opening met the ADIRS requirements, the suction tubing was patent, and the registration holes on the attachment surface were intact (Fig. 1E–G).

### Surgical procedure

**Fig. 3:**
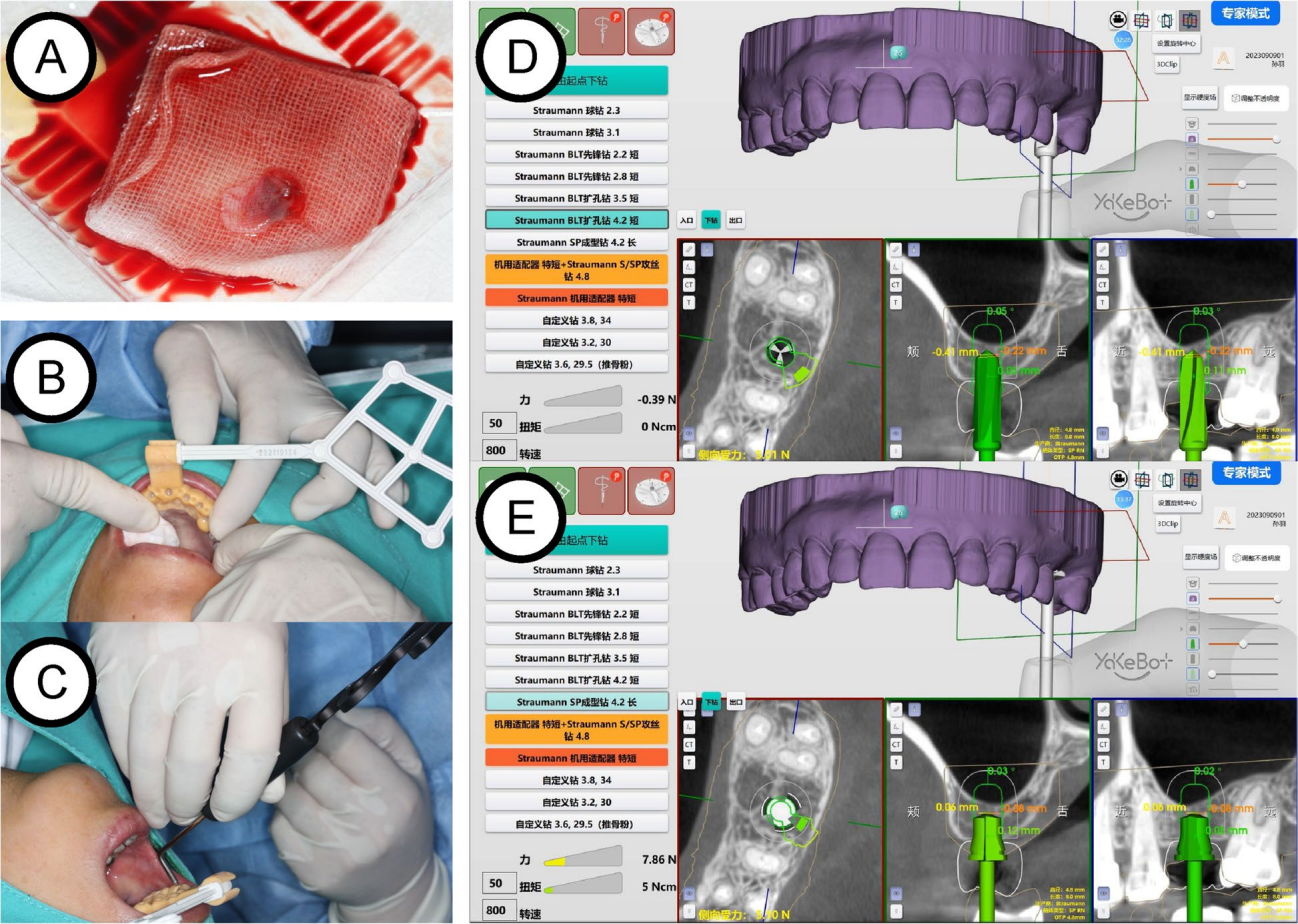
Intraoperative registration and stepwise osteotomy. **A** Prepared CGF membrane. **B** Try-in and fixation of the positioning guide using intraoral markers. **C** Spatial and guide registration to transfer the preoperative plan to the patient. **D** Confirmation and input of the final drilling endpoint. **E** Cervical-shaping drilling to prepare the smooth-neck region of the implant

**Fig. 4:**
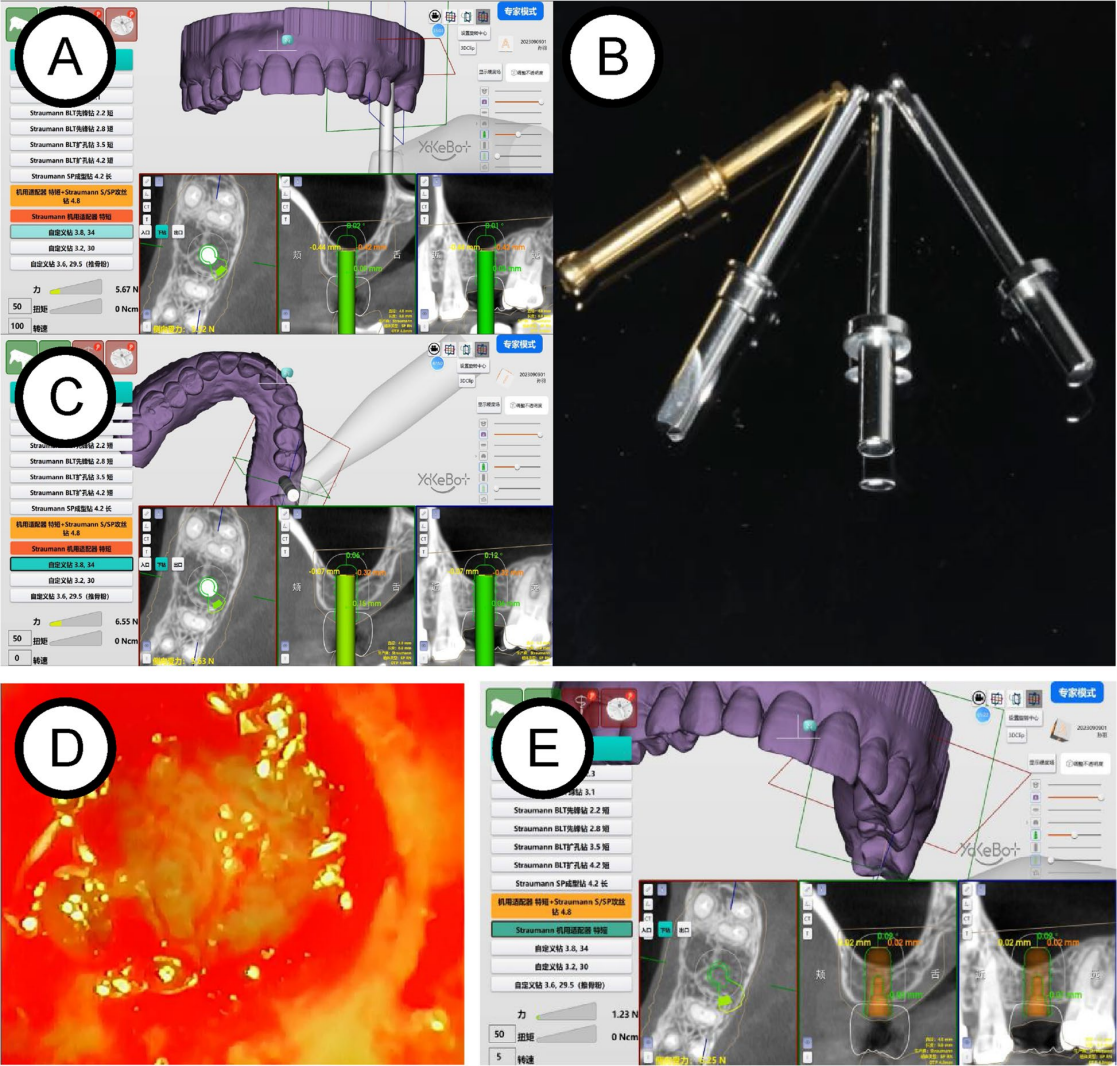
Key ADIRS-assisted TSFE steps. **A** Gentle polishing of the residual cortical bone at the sinus floor. **B** Self-designed sinus elevation instrument set: left two, grinding instruments for the sinus floor; right two, membrane-elevating instruments. **C** Gradual elevation of the Schneiderian membrane in TSFE mode. **D** Endoscopic inspection of Schneiderian membrane integrity through the implant osteotomy, with visualization of membrane morphology and surface vessels. **E** Robotic interface display showing the definitive implant position and insertion trajectory

Two tubes of venous blood were drawn and processed in a concentrated growth factor (CGF) centrifuge (Medifuge, Italy) for 17 min to obtain CGF membranes for sinus grafting (Fig. 3A). The positioning guide and intraoral markers were disinfected in 75% alcohol for 15 min. After extraoral and intraoral disinfection and draping, local infiltration anesthesia was achieved with 2% mepivacaine. The guide was temporarily bonded to the anterior maxilla and connected to the intraoral marker (Fig. 3B). Robotic arm calibration, spatial registration, and guide registration were then performed to transfer the preoperative plan to the patient (Fig. 3C). Under ADIRS guidance, stepwise osteotomy was performed, and the final drilling endpoint was set 1 mm short of the sinus floor to preserve a 1 mm bony plate (Fig. 3D). A cervical-shaping bur was used to prepare the smooth-neck region of the implant site as planned (Fig. 3E). The system was then switched to TSFE mode. A self-designed sinus floor grinding instrument was used to gently polish the residual cortical bone at the sinus floor (Fig. 4A). According to the planned trajectory, an appropriate membrane-elevating instrument with a smooth, round tip was selected to elevate the Schneiderian membrane gradually (Fig. 4B, C).

Endoscopic inspection (performed with a separate dental digital observation endoscope, Bangvo P series, China) combined with the Valsalva maneuver was used to confirm membrane integrity (Fig. 4D). A collagen bone substitute (Geistlich Pharma, Switzerland), trimmed to size, together with the CGF membrane, was delivered through the osteotomy while avoiding excessive pressure on the membrane. The implant was then placed at the preplanned position (Fig. 4E). If the insertion torque was < 30 N·cm, a cover screw was installed.

### Postoperative care

Postoperative CBCT was obtained within 24 h after surgery to assess implant position and sinus status. Patients received prophylactic antibiotics (cephradine 250 mg plus metronidazole 200 mg, three times daily for 5 days) and analgesics (paracetamol 500 mg as needed). From 24 h postoperatively, patients rinsed with 0.12% chlorhexidine solution three times daily for 7 days. Patients were instructed to avoid forceful nose blowing, sneezing with the nose closed, chewing hard foods, diving, or air travel for 2 weeks.

### Outcome measures

Preoperative virtual implant models and postoperative CBCT datasets were superimposed in the software bundled with the robotic system. The following deviations between planned and actual implant positions were calculated: (1) three-dimensional global deviation at the coronal and apical levels; (2) horizontal components (facial–lingual and mesio–distal) and the vertical component of the deviation; and (3) angular deviation between the long axes of the implants. Linear deviations were reported in millimeters, and angular deviations were reported in degrees. Intraoperative sinus membrane perforations—confirmed endoscopically and by the Valsalva maneuver—and any other surgery-related complications were also documented.

### Statistical analysis

Statistical analysis was performed using IBM SPSS Statistics 27.0 (IBM Corp., Armonk, NY, USA). Continuous variables were expressed as mean ± standard deviation and, when appropriate, as median, interquartile range, minimum, maximum, and 95% confidence intervals. Normality was assessed with the Shapiro–Wilk test. Depending on data distribution, Student’s t test or the Mann–Whitney U test was used to compare outcomes between the flat and sloped groups. Additional analyses and graphical visualization were conducted in R software (version 4.2.2). To improve the transparency of non-significant findings, a post hoc sensitivity analysis was performed after data collection. Based on Welch’s two-sample t test (unequal variances, two-sided α = 0.05) and using the observed sample sizes, within-group standard deviations, standard errors, and Welch–Satterthwaite degrees of freedom, we estimated the minimum detectable differences (MDDs) for target powers of 0.10, 0.30, 0.50, 0.80, and 0.90 under a noncentral t distribution.

## Results

### Demographic and surgical characteristics of the participants

This retrospective cohort included 11 patients (7 males and 4 females) who underwent ADIRS-assisted TSFE, with 12 implants placed in total (one patient received two implants). The mean age was 59.45 ± 13.16 years (range, 33–74). All implants were placed in the posterior maxilla, comprising one premolar and eleven molar sites. The mean RBH before surgery was 6.14 ± 0.78 mm. Seven implant sites were classified as having a flat sinus floor and five implant sites as having a sloped sinus floor. The mean insertion torque at implant placement was 32.92 ± 3.34 N·cm. Detailed demographic and surgical characteristics for each implant are presented in Table 1.

**Table 1:** Participants’ demographic and surgical information

Implant No	Patient No	Age (yrs)	Sex	Site	Residual bone height (mm)	Sinus lift height (mm)	Sinus floor inclination	Implant parameters	Insertion Torque (N.cm)
1	I	56	M	16	4.95	3.20	Flat	Straumann SP RN 4.8*8	35
2	II	72	M	16	5.75	1.35	Flat	Straumann SP RN 4.8*8	25
3	III	74	F	16	6.75	1.20	Flat	Straumann SP RN 4.8*8	35
4	IV	66	F	26	5.70	1.65	Flat	Straumann SP RN 4.8*8	35
5	V	56	M	26	6.22	4.65	Flat	Straumann BLT RC 4.8*10	35
6	VI	33	F	16	6.60	3.60	Flat	Straumann SP RN 4.1*8	35
7	VII	74	M	16	7.80	1.20	Flat	Straumann SP RN 4.8*8	35
8	VII	74	M	26	5.25	6.16	Sloped	Straumann SP RN 4.1*10	35
9	VIII	52	M	15	5.40	4.95	Sloped	Straumann SP RN 4.1*10	30
10	IX	43	F	26	6.45	6.15	Sloped	Straumann SP RN 4.1*10	30
11	X	67	M	26	6.45	3.90	Sloped	Straumann SP RN 4.1*10	30
12	XI	61	M	16	6.38	5.85	Sloped	Straumann SP RN 4.1*10	35

*Abbreviations: N·cm* newton centimeter, *SP* Standard Plus, *RN* Regular Neck, *BLT* Bone Level Tapered, *RC* Regular CrossFit

### Overall trueness of implant placement and complications

**Fig. 5:**
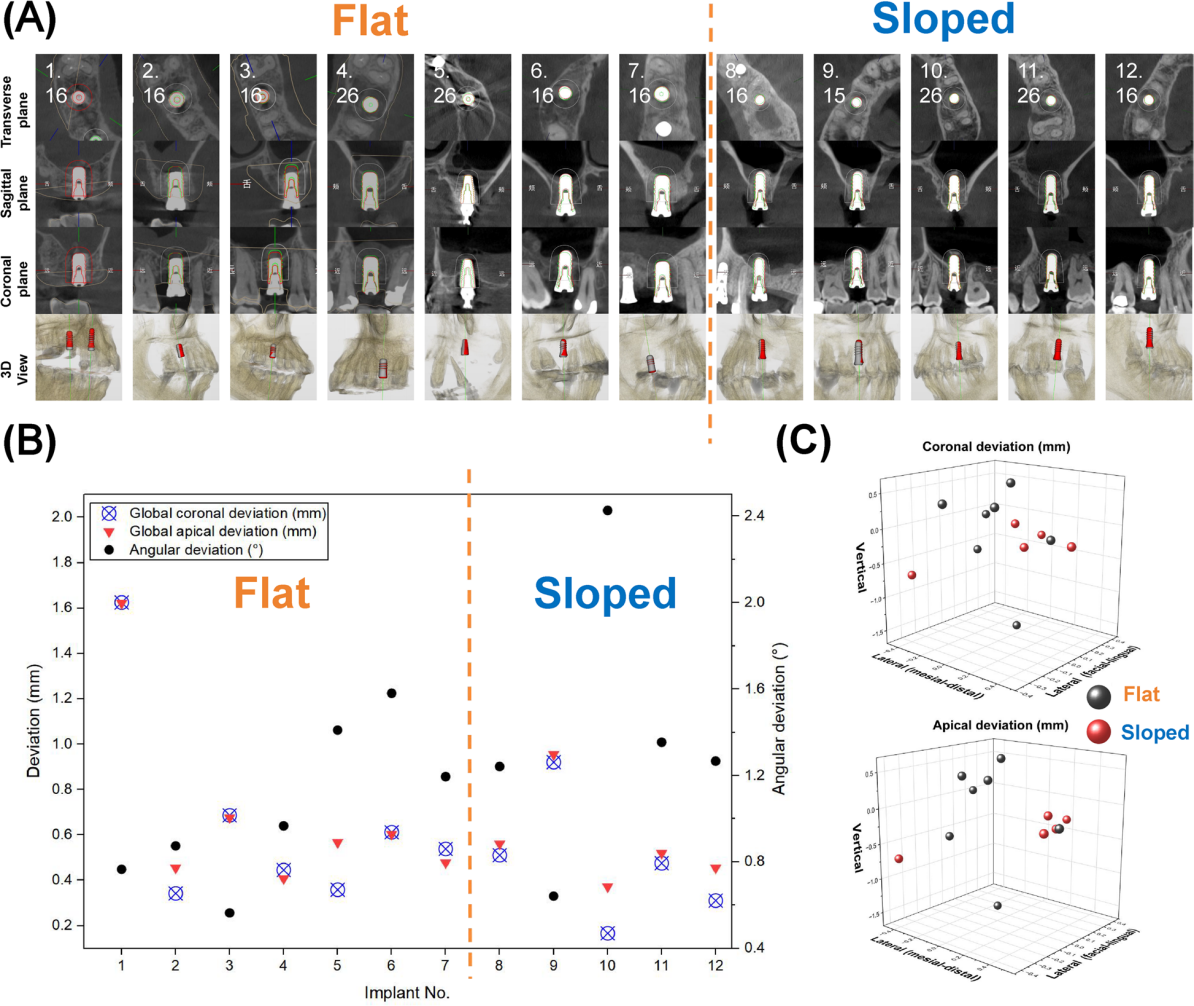
Postoperative trueness assessment. **A** CBCT fusion of planned (green) and placed (red) implants, grouped by sinus floor morphology (Flat vs Sloped). **B** Global coronal, apical, and angular deviations for each implant. **C** 3D scatterplots of lateral and vertical deviations. Sign convention: facial (+), lingual (−); distal (+), mesial (−); shallower (+), deeper (−)

Planned and actual implant positions showed close agreement (Fig. 5). Across the 12 implants, the mean global deviations at the coronal and apical levels were 0.58 ± 0.38 mm (range, 0.17–1.63; 95% CI, 0.34–0.83) and 0.64 ± 0.35 mm (range, 0.37–1.62; 95% CI, 0.42–0.86), respectively. The mean angular deviation was 1.19 ± 0.50° (range, 0.56–2.43; 95% CI, 0.87–1.51).

At the coronal level, the mean lateral deviations were 0.16 ± 0.11 mm in the facial–lingual direction (range, 0.03–0.37; 95% CI, 0.09–0.23) and 0.21 ± 0.17 mm in the mesio–distal direction (range, 0.01–0.50; 95% CI, 0.10–0.31). At the apical level, the corresponding values were 0.15 ± 0.11 mm (range, 0.01–0.36; 95% CI, 0.08–0.22) and 0.29 ± 0.19 mm (range, 0.01–0.56; 95% CI, 0.18–0.41), respectively. Vertical deviations were 0.43 ± 0.45 mm at the coronal level (range, 0.01–1.62; 95% CI, 0.15–0.72) and 0.43 ± 0.45 mm at the apical level (range, 0.02–1.61; 95% CI, 0.15–0.71). Shapiro–Wilk testing indicated that most variables approximated normal distributions (*p* > 0.05). Detailed descriptive statistics are summarized in Table 2. No intraoperative or postoperative complications occurred, and endoscopic and Valsalva examinations were negative for Schneiderian membrane perforation in all cases.

**Table 2:** Summary statistics of implant deviations

Metric	Global deviation (mm)		Lateral deviation (mm)				Vertical deviation (mm)		Angular deviation (°)
	**Coronal**	**Apical**	**Coronal** **(facial-lingual)**	**Coronal** **(mesial-distal)**	**Apical** **(facial-lingual)**	**Apical** **(mesial-distal)**	**Coronal**	**Apical**	
Mean ± SD	0.58 ± 0.38	0.64 ± 0.35	0.16 ± 0.11	0.21 ± 0.17	0.15 ± 0.11	0.29 ± 0.19	0.43 ± 0.45	0.43 ± 0.45	1.19 ± 0.50
Min	0.17	0.37	0.03	0.01	0.01	0.01	0.01	0.02	0.56
P25	0.35	0.46	0.05	0.05	0.09	0.12	0.08	0.08	0.79
Median	0.49	0.54	0.16	0.13	0.12	0.33	0.35	0.35	1.22
P75	0.67	0.66	0.22	0.34	0.19	0.45	0.63	0.63	1.40
Max	1.63	1.62	0.37	0.50	0.36	0.56	1.62	1.61	2.43
Lower 95% CI	0.34	0.42	0.09	0.10	0.08	0.18	0.15	0.15	0.87
Upper 95% CI	0.83	0.86	0.23	0.31	0.22	0.41	0.72	0.71	1.51
Shapiro–Wilk test (*p*)	0.7984 (0.0090)	0.6897 (0.0007)	0.9304 (0.3847)	0.8900 (0.1178)	0.8729 (0.0711)	0.9474 (0.5991)	0.8229 (0.0172)	0.8233 (0.0175)	0.9050 (0.1842)

*Abbreviations: SD* standard deviation, *Min* minimum, *P25* 25th percentile, *P75* 75th percentile, *Max* maximum, *CI* confidence interval

### Comparison between flat and sloped sinus floor groups

Between-group comparisons are shown in Table 3. None of the trueness metrics differed significantly between the flat (*n* = 7) and sloped (*n* = 5) groups (all *p* > 0.05).

**Table 3:** Comparison of trueness metrics between flat and sloped groups

Parameters	Flat group (*n* = 7)	Sloped group (*n* = 5)	*p* (inter-group)^†^
Global deviation (mm, mean ± SD)			
Coronal	0.66 ± 0.44	0.48 ± 0.28	0.4318
Apical	0.69 ± 0.42	0.57 ± 0.23	0.5581
Lateral deviation (mm, mean ± SD)			
Coronal (facial-lingual)	0.15 ± 0.09	0.17 ± 0.13	0.6566
Coronal (mesial-distal)	0.19 ± 0.12	0.23 ± 0.24	0.7303
Apical (facial-lingual)	0.10 ± 0.05	0.21 ± 0.14	0.1035
Apical (mesial-distal)	0.23 ± 0.20	0.38 ± 0.12	0.1545
Vertical deviation (mm, mean ± SD)			
Coronal	0.53 ± 0.53	0.29 ± 0.28	0.3885
Apical	0.53 ± 0.53	0.29 ± 0.29	0.3833
Angular deviation (°, mean ± SD)	1.05 ± 0.36	1.39 ± 0.65	0.2752

*Abbreviation: SD* standard deviation

^†^*p* (between-group) values represent the differences between the flat and sloped groups

Supplementary Table S1 reports the MDDs for the prespecified power levels (0.10–0.90). At 80% power, the detectable differences were approximately 0.66 mm for global coronal deviation, 0.59 mm for global apical deviation, 1.09° for angular deviation, 0.23 mm (facial–lingual) and 0.40 mm (mesio–distal) for lateral coronal deviations, 0.24 mm (facial–lingual) and 0.29 mm (mesio–distal) for lateral apical deviations, and 0.74 mm and 0.75 mm for vertical coronal and apical deviations, respectively. Because the observed group differences were all smaller than these thresholds, the non-significant findings are more likely attributable to limited sample size and statistical power than to a true absence of morphology-related effects.

## Discussion

Loss of posterior maxillary teeth is common in clinical practice [1, 17, 18]. Following tooth loss, the absence of masticatory stimulation and the effect of respiratory negative pressure contribute to progressive vertical ridge resorption and maxillary sinus pneumatization, resulting in inadequate residual bone height for implant placement [1, 17, 19]. In this context, TSFE has become an important approach to facilitate implant rehabilitation under limited residual bone height [1, 2, 17, 20]. However, conventional TSFE techniques, such as osteotomes and other manual instruments, provide limited precision in locating the sinus floor and controlling the elevation depth, and outcomes remain highly operator dependent [1, 2, 6, 21]. Furthermore, restricted intraoperative visibility within the sinus increases the risk of Schneiderian membrane perforation, which has been reported in approximately 3–8% of cases and may lead to graft migration, sinusitis, or other postoperative complications [1, 5, 6, 19, 21].

To enhance the precision and safety of TSFE, computer-assisted approaches, including static surgical guides and dynamic navigation systems, have been introduced [6–8, 12]. Static guides are difficult to modify once fabricated; when anatomic discrepancies or guide misfit occur, the possibilities for intraoperative adjustment are limited. Extensive guide coverage may also impair irrigation and visualization, thereby increasing the risk of thermal or mechanical injury to the bone [6, 7]. Dynamic navigation offers real-time visualization and greater flexibility, but drill trajectory and depth are still manually controlled according to screen feedback, so trueness and reproducibility remain largely dependent on operator experience [6, 7, 12].

Robot-assisted computer-aided implant surgery (r-CAIS) establishes a closer linkage between virtual planning and intraoperative execution by providing direct visualization and active control of implant trajectory and position. In the TSFE setting, small case series using semi-active robotic systems have reported mean global coronal, global apical, and angular deviations of 0.50–0.73 mm (SD 0.20–0.35 mm), 0.54–0.78 mm (SD 0.21–0.31 mm), and 1.25–2.20° (SD 0.61–2.39°), respectively, with Schneiderian membrane perforation rates of 0–2.5% (most series reporting none) [4, 8, 9, 12–14]. All of these data were generated using the Remebot platform, whereas clinical evidence for the YakeBot-based ADIRS in TSFE remains limited. To date, only one retrospective case series has described ADIRS-assisted TSFE, reporting mean global coronal, global apical, and angular deviations of 0.90 ± 0.58 mm, 1.01 ± 0.60 mm, and 2.29 ± 1.28°, respectively, without Schneiderian membrane perforation in 17 patients [9]. Our current pilot cohort adds to this emerging body of evidence, demonstrating that integrating workflow-level refinements—such as custom stop-limited instruments and stricter endpoint control—yields promising preliminary positional trueness and operational safety.

In this study, we systematically delineated the ADIRS workflow for TSFE and conducted a preliminary evaluation of its trueness and safety. Across 12 implants, the mean global coronal and apical deviations were 0.58 ± 0.38 mm and 0.64 ± 0.35 mm, respectively, and the mean angular deviation was 1.19 ± 0.50°. No Schneiderian membrane perforations were observed. These findings suggest that ADIRS can achieve favorable positional trueness with a reassuring safety profile in TSFE [9, 12, 15, 16].

**Fig. 6:**
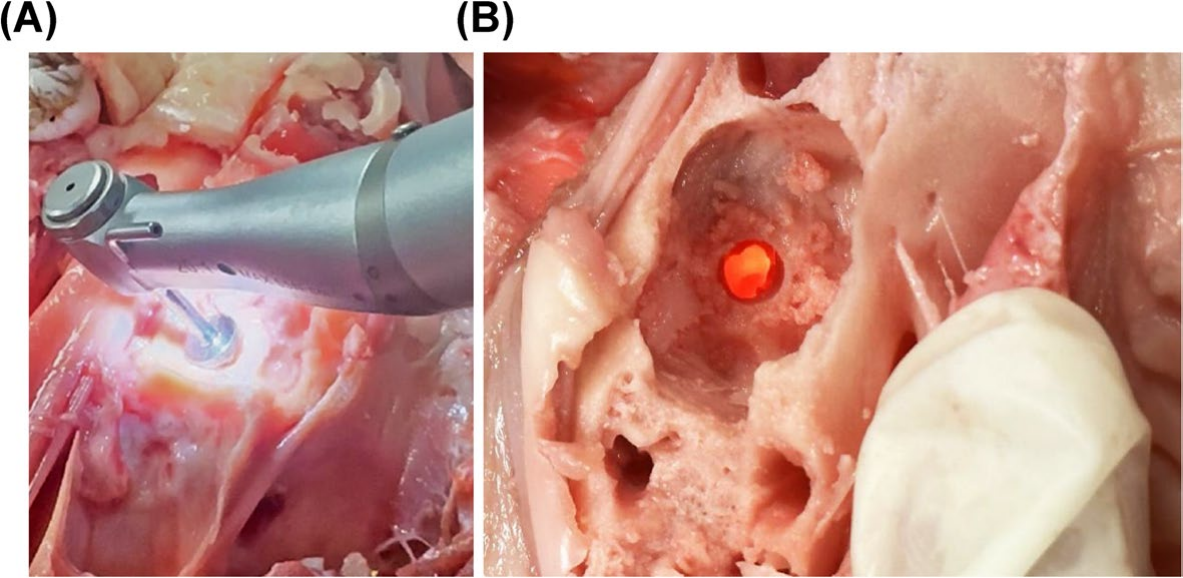
Preclinical animal-model validation of the self-designed sinus elevation instruments. **A** Elevation of the maxillary sinus membrane using the custom instrument set. **B** Intact sinus membrane after elevation

Schneiderian membrane perforation is the most common complication of TSFE [5, 19, 21]. To simplify the workflow, mitigate perforation risk, and reduce intraoperative manipulation, we developed a novel sinus elevation instrument. The device incorporates a stop ring (Fig. 4B) that provides a mechanical limit, thereby reducing inadvertent over-advancement and the associated risk of membrane injury during robot-guided transcrestal elevation. Its blunt, shallow-concave tip is designed to distribute contact pressure during submembrane expansion, further lowering the risk of mechanical trauma from residual thin bony lamellae to the Schneiderian membrane [2, 22, 23]. In TSFE, the morphology of the maxillary sinus floor is a key factor influencing perforation risk. Compared with flat sinus floors, sloped sinus floors are generally considered more prone to perforation [19, 21, 24]. Before clinical application, feasibility was assessed in a preclinical porcine-head cadaver model series, in which use of the custom elevator achieved more than 4 mm of sinus membrane elevation while maintaining membrane integrity (Fig. 6).

Finally, we compared implant placement trueness between sloped and flat sinus floors under ADIRS guidance. Given the non-significant between-group differences, we adopted a cautious interpretation. With the present sample size and within-group variability, the study had adequate power to detect moderate or larger effects but was underpowered to detect small effects. Post hoc sensitivity analyses showed that, at a two-sided α of 0.05 and 80% power, the MDDs exceeded the observed differences between groups. These results suggest that the absence of statistical significance is more likely due to limited power than to true equivalence. It is also important to note that the MDD is distinct from the minimum clinically important difference (MCID): the MDD is determined by sample size and variance, whereas the MCID is defined by clinical relevance. If future studies aim to detect smaller differences, for example, on the order of 0.2 mm, they should increase the planned sample size based on the baseline variance data and MDDs established in this pilot study, and further reduce measurement variability through standardized instruments and workflow optimization, in order to lower the MDD, increase power, and reduce the risk of false-negative conclusions [6, 9, 19, 24].

This study has several limitations. First, it was a single-center retrospective study with a limited sample size, and the risks of selection bias and residual confounding cannot be excluded. Second, the absence of a comparator group (e.g., freehand surgery, static guides, or dynamic navigation) restricts direct benchmarking and limits the ability to contextualize the trueness performance of ADIRS. Third, our analyses focused on short-term placement trueness and intraoperative safety, and clinically important outcomes such as implant survival, marginal bone loss, and long-term sinus health were not evaluated. Taken together, the results should therefore be interpreted with appropriate caution [9, 12, 18, 19, 24].

## Conclusion

Within the limitations of this pilot retrospective study and its small sample size, ADIRS-assisted TSFE demonstrated stable positional trueness with no Schneiderian membrane perforation observed. Multicenter, prospective comparative studies are warranted to confirm its clinical value and evaluate long-term outcomes.

## Supplementary Information

Supplementary Material 1.

## Data Availability

The datasets generated and/or analysed during the current study are not publicly available due to patient privacy and institutional data protection policies but are available from the corresponding author on reasonable request.

## Abbreviations

ADIRS: Autonomous dental implant robotic system (YakeBot-based)
BLT: Bone Level Tapered (Straumann implant)
CAIS: Computer-assisted implant surgery
CBCT: Cone-beam computed tomography
CGF: Concentrated growth factor
DASK: Dentium Advanced Sinus Kit
DRS-FT250: YakeRobot DRS-FT250 robotic arm
IBM: International Business Machines (vendor of SPSS)
MCID: Minimum clinically important difference
MDD: Minimum detectable difference
r-CAIS: Robot-assisted computer-assisted implant surgery
RBH: Residual bone height
RC: Regular CrossFit (Straumann platform)
RN: Regular Neck (Straumann tissue-level platform)
SP: Standard Plus (Straumann tissue-level implant)
SPSS: Statistical Package for the Social Sciences
STROBE: Strengthening the Reporting of Observational Studies in Epidemiology
TRIOS: 3Shape TRIOS intraoral scanner
TSFE: Transcrestal maxillary sinus floor elevation
